# Assembly of Genome and Resequencing Provide Insights into Genetic Differentiation between Parents of Hulong Hybrid Grouper (*Epinephelus fuscoguttatus* ♀ *× E. lanceolatus* ♂)

**DOI:** 10.3390/ijms241512007

**Published:** 2023-07-26

**Authors:** Yang Yang, Leilei Zeng, Tong Wang, Lina Wu, Xi Wu, Junhong Xia, Zining Meng, Xiaochun Liu

**Affiliations:** 1 State Key Laboratory of Biocontrol, Institute of Aquatic Economic Animals and Guangdong Provincial Key Laboratory for Aquatic Economic Animals, Life Sciences School, Sun Yat-sen University, Guangzhou 510275, China; yangy888@mail.sysu.edu.cn (Y.Y.); zlei5716@163.com (L.Z.); sysuwangtong@163.com (T.W.); wuln5@mail2.sysu.edu.cn (L.W.); wuxi577@126.com (X.W.); xiajunh3@mail.sysu.edu.cn (J.X.); 2 Key Laboratory of Tropical Marine Fish Germplasm Innovation and Utilization, Ministry of Agriculture, Sanya 572025, China; 3 Key Laboratory of Tropical Aquatic Germplasm of Hainan Province, Sanya 572025, China; 4 Southern Laboratory of Ocean Science and Engineering, Zhuhai 519000, China

**Keywords:** grouper, genome, SNP, structural variation, growth traits

## Abstract

The Hulong hybrid grouper was bred from the brown-marbled grouper (*Epinephelus fuscoguttatus*) *♀* and the giant grouper (*E. lanceolatus*) ♂, combining the advantageous traits of both parents. Possessing an excellent performance, this hybrid’s cultivation promotes the development of the grouper industry. Its male parent, the giant grouper, possesses the fastest growth and the largest body size among all coral-reef-dwelling fish. This species is not only an economically important species in marine aquaculture, but it is also an ideal male parent in the interspecific crossing of grouper species. In the present study, a high-quality chromosome-level genome of the giant grouper was constructed with a total length of 1.06 Gb, consisting of 24 chromosomes and 69 scaffolds. To analyze the genetic differences between the parents of the Hulong hybrid grouper, the structural variations (SVs) between both parental genomes were detected, and a total of 46,643 SVs were obtained. High-quality SNPs were identified from resequencing data. There were significant differences between the two genomes, and the average *F*_ST_ reached 0.685. A total of 234 highly differentiated regions were detected with an *F*_ST_ > 0.9. The protein-coding genes involved in SVs and highly differentiated regions were significantly enriched in metabolic pathways, including fatty metabolism, carbohydrate metabolism, amino acid metabolism and the TCA cycle. These genes may be related to the differences in feeding preferences and the ability to digest carbohydrates between the two grouper species under natural conditions. In addition, protein-coding genes related to the cell cycle and p53-signaling pathway were also detected. These genes may play important roles in the regulation of body size and growth performance. This research provides genomic resources for further breeding works and evolutionary analyses.

## 1. Introduction

Groupers (Epinephelidae, perciformes) are coral-reef-dwelling fish species that are mostly distributed in the tropical and temperate marine areas. Groupers include more than 160 species in 16 genera [1], and, among them, approximately 47 grouper species have been cultured [2]. Groupers are popular in the markets of South and Southeast Asia due to their high nutrient value, tender meat and delicious taste. Due to the incomplete reproductive isolation and various grouper species, crossbreeding is the most common tool in the breeding of groupers, and many new hybrids have been obtained by means of artificial insemination. Among the hybrids, brown-marbled grouper (*Epinephelus fuscoguttatus*) ♀ × giant grouper (*E. lanceolatus*) ♂ is the most successful hybrid grouper. It possesses an excellent growth performance and strong stress resistance, and it greatly promotes the development of the grouper industry [3]. At present, the annual output of this hybrid accounts for more than 70% of the grouper market in China [4].

The giant grouper is the male parent of the Hulong hybrid grouper, and it is the largest grouper species in the world. Its length can reach 2.7 m and its weight can reach 400 kg [5]. The giant grouper has been widely applied as the male parent in other interspecific crossings of grouper species due to its excellent growth traits. To date, at least five hybrids, including kelp grouper (*E. moara*) ♀ × giant grouper ♂ [6], orange-spotted grouper (*E. coioides*) ♀ × giant grouper ♂ [7], brown-marbled grouper ♀ × giant grouper ♂ [8], humpback grouper (*Cromileptes altivelis*) ♀ × giant grouper ♂ [9] and red-spotted grouper (*E. akaara*) ♀ × giant grouper ♂ [10], have been reported. The growth traits of these hybrid groupers are all significantly higher than those of their female parents. Obviously, the genome of the giant grouper plays an important role in the growth advantage of hybrid groupers. The giant grouper and brown-marbled grouper are the parents of the Hulong hybrid grouper; however, there are significant differences in their sizes: the brown-marbled grouper weighs 4~12 kg, while the giant grouper can reach 400 kg. Analyses of the growth difference between the two grouper species contribute to understanding the genetic basis for growth advantages in giant groupers. Recent research reported some genetic analyses of the brown-marbled grouper and the giant grouper; high levels of genome synteny were revealed, and their divergence time was 10.49–22.50 Myr, which are considered pivotal factors for the success of hybrid groupers [11]. An F2 segregation population of the Hulong hybrid grouper was applied to analyze the genetic basis of the growth advantage, and several genes involved in energy metabolism, development, stress tolerance, the immune response and visual perception were found to be associated with growth traits in the juvenile stage of the giant grouper [12].

In order to provide useful genetic information for the conservation and breeding of the giant grouper, a high-quality chromosome-level genome is essential. Although two genomes of the giant grouper have been assembled recently [4,13], there were some flaws in them. For Zhou et al.’s assembled genome, they only applied Illumina sequencing data, the contig N50 value was only 119.9 kb and the number of assembled scaffolds was 4207. The numerous gaps and fragments in the giant grouper genome destroy the integrity and lower the correctness of the genome, and hamper efforts at locating trait-related genes and markers. In Wang et al.’s assembled genome, the pseudochromosomes of the giant grouper were constructed based on the genetic linkage map of another grouper (orange-spotted grouper), where only 39.41% and 88.62% of the scaffolds and bases, respectively, could be anchored to pseudochromosomes. Thus, the genome of the giant grouper needs to be further improved.

In the present study, first, we assembled a high-quality chromosome-level giant grouper genome using the PacBio platform and Hi-C technology, and we predicted the gene structures; second, genomic differences between the giant grouper and the brown-marbled grouper were detected. These genetic differences were related to feeding preferences, the ability to digest carbohydrates and the cell cycle. This research provides a better genome and insights into the genetic differentiation between the brown-marbled grouper and giant grouper.

## 2. Results

### 2.1. Genome Assembly

The genome characteristics of the giant grouper were predicted using k-mer methods with Illumina data. A total of 169.44 Gb of high-quality data with Q20 > 96.99% and Q30 > 92.27% were obtained (Table S1). The heterozygosity and genome size were approximately 0.07% and 1.08 Gb, respectively. The repeat-sequence ratio and GC ratio were 26.08% and 41.20%, respectively (Figure S2).

A total of 134.42 Gb of PacBio-sequencing clean data were obtained, and the average length and N50 of the reads were 18.55 Kb and 29.14 Kb, respectively (Table S1). The assembled genome of the giant grouper was 1.06 Gb in length with a contig N50 of 24.33 Mb and 170 contigs. The integrality of the assembled genome was verified using BWA, CEGMA and BUSCO. A total of 99.31% of the Illumina clean reads were mapped onto the assembled genome, and, among them, 98.21% were properly mapped (Table S2). The CEGMA results show that 438 (95.63%) CEGs were present in the assembled genome, as well as 188 (75.81%) highly conserved CEGs (Table S3). The results from the BUSCO alignment showed that the assembled genome contained 4463 (97.36%) complete BUSCOs, among which 4357 (95.05%) were single-copy BUSCOs (Table S4).

### 2.2. Pseudochromosome Construction

**Figure 1:**
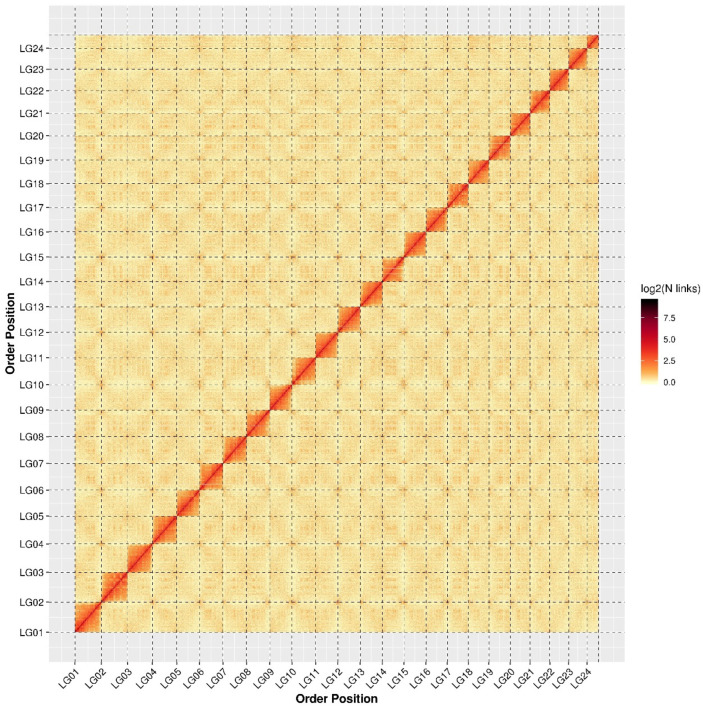
Heatmap of interaction of contigs for giant grouper genome assemblies. A deeper color indicates a higher interaction level of contigs. Red indicates a high interaction level and white indicates a low interaction level.

A total of 1.06 Gb (99.96%) and 145 (81.46%) contigs were anchored into 24 chromosomes using Hi-C technology (Table S5 and Figure 1). Among the anchored contigs, 133 (91.72%) were anchored in the proper order. Ultimately, a total of 69 scaffolds were obtained, and N50 and N90 were 45,206,398 bp and 40,025,893 bp in length, respectively.

### 2.3. Genome Annotation

Non-coding RNAs, including micro-RNAs (miRNAs), ribosomal RNAs (rRNAs) and transfer RNAs (tRNAs), were predicted based on the Rfam database. In total, 835 miRNAs, 190 rRNAs and 1480 tRNAs were obtained. In addition, 1186 pseudogenes were predicted using GeneWise software [14].

In the giant grouper genome, a total of 1,000,756 repeat sequences were predicted with 322,857,005 bp, which accounted for 30.34% of the whole genome. Terminal inverted repeats (TIRs) were the largest group in the repeat sequences, and they included 480,765 sequences and accounted for 17.42% of the genome. We also detected 3074 simple-sequence repeats (SSRs), which accounted for 0.09% of the genome (Table S6).

Gene structures were predicted using ab initio, homologous sequencing and RNA-seq. Ultimately, 28,313 genes (Table S7 and Figure S3) were predicted, with an average gene length of 16,339.58 bp and an average exon length of 2650.08 bp (Table S8). A total of 27,107 (95.74%) genes were annotated based on annotation databases: 27,057 (95.56%) of them were mapped into the NR database, while 26,692 (94.27%) were mapped into the TrEMBL database (Table S9).

### 2.4. Analysis of Differences in Genomic Structure between the Brown-Marbled Grouper and Giant Grouper

A total of 46,643 structural variations were detected between the brown-marbled grouper and giant grouper genomes, including 4539 insertions with a total length of 1,661,757 bp, 3577 deletions with a total length of 1,035,628 bp, 171 tandem expansions with a total length of 460,709 bp, 5 tandem contractions with a total length of 6247 bp, 22,188 repeat expansions with a total length of 31,273,681 bp and 16,163 repeat contractions with a total length of 17,907,672 bp (Table S10). The variant-size histogram of the structural variations in the genomes shows that the sizes of the sequences are inversely proportional to their mutation ratios (Figure S4).

**Figure 2:**
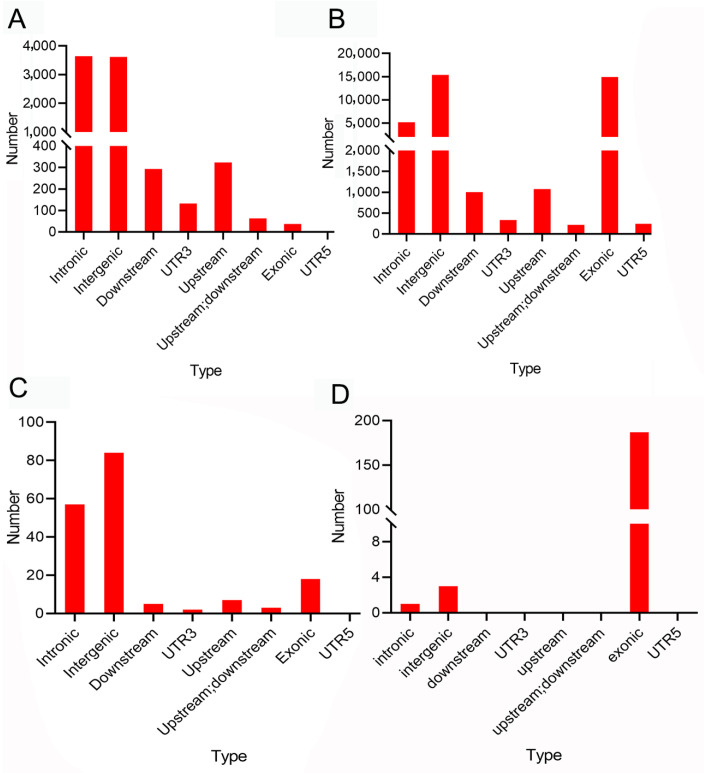
Distribution of structural variation in genomes between brown-marbled grouper and giant grouper: (**A**) annotation of insertion and deletion; (**B**) annotation of repeat expansion and contraction; (**C**) annotation of tandem expansion and contraction; (**D**) annotation of genomic inversion.

Based on annotations using Annovar software, deletions and insertions were mainly located in introns and intergenic regions (3648 and 3619, respectively). A total of 37 deletions and insertions were located in exons. Repeat mutations were mainly located in intergenic regions and exons (15,382 and 14,913, respectively). Tandem expansions and contractions were mainly located in intergenic regions (Figure 2).

**Figure 3:**
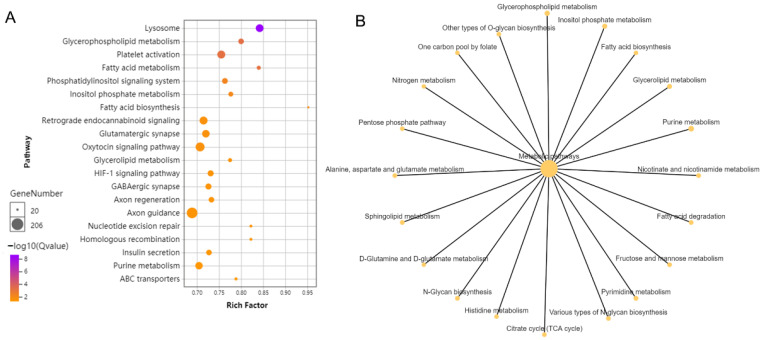
KEGG enrichment of protein-coding genes containing repeat mutations between brown-marbled grouper and giant grouper genomes. (**A**) Bubble pattern of KEGG enrichment analysis. The sizes of the bubbles indicate the numbers of genes, purple indicates a high enrichment level and orange indicates a low enrichment level. (**B**) Metabolism pathway network of protein-coding genes containing repeat mutations. The size of the bubble indicates the gene’s number of pathways, and the color of the bubble indicates the enrichment degree of the pathways.

The protein-coding genes that possessed repeat mutations in exons were enriched based on the KEGG database. These protein-coding genes were significantly enriched into metabolic pathways (Figure 3A), including lipid metabolism (glycerophospholipid metabolism, fatty acid metabolism and biosynthesis), amino acid metabolism (histidine, glutamate, alanine, aspartate and glutamate metabolism), glycometabolism (insulin secretion, fructose and mannose metabolism, N-glycan metabolism) and the TCA cycle (Figure 3B). In addition, some genes involved in the lysosome, HIF-1 signaling pathway, glycan metabolism and fructose and mannose metabolism were also enriched.

### 2.5. Analysis of Genetic Differentiation between the Brown-Marbled Grouper and Giant Grouper

**Figure 4:**
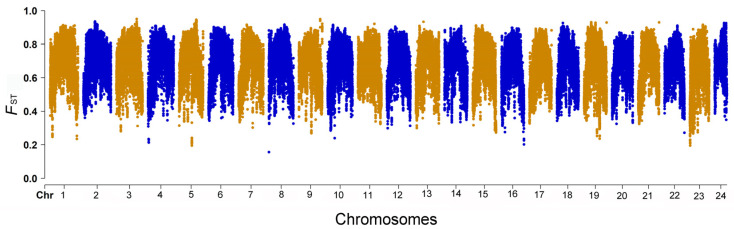
Manhattan plot of *F*_ST_ values of SNPs between giant grouper and brown-marbled grouper genomes.

**Figure 5:**
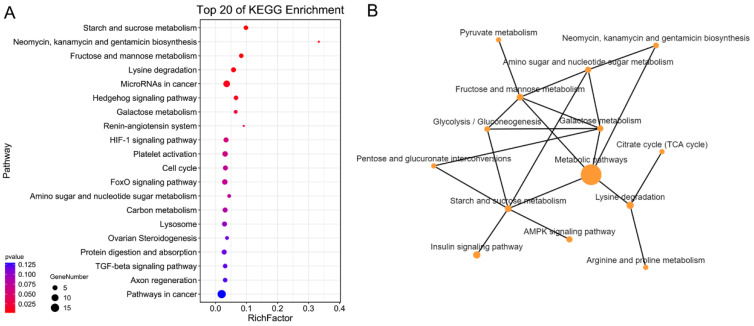
KEGG enrichment of protein-coding genes involved in highly differentiated regions between brown-marbled grouper and giant grouper genomes. (**A**) Gradient of KEGG enrichment. The sizes of bubbles indicate the numbers of genes, purple indicates a high enrichment level and orange indicates a low enrichment level. (**B**) Metabolism pathway network. The size of the spot indicates the gene’s number of pathways.

A total of 3,611,851 SNPs were detected between the giant grouper and brown-marbled grouper genomes. The density of the SNPs was 293.59 bp/SNP. The SNP density of Chromosome 24 was the highest, reaching 233.94 bp/SNP, whereas the SNP density of Chromosome 18 was the lowest, at 332.41 bp/SNP. A genetic differentiation analysis between the two groupers was carried out. There were significant differences between the two genomes, and the average *F*_ST_ reached 0.685. The average *F*_ST_ of Chromosome 5 was the lowest, with a value of 0.674, whereas the average *F*_ST_ of Chromosome 2 was the highest, reaching 0.701. *F*_ST_ values were lower at both ends of each chromosome compared to the middle parts. A total of 234 regions with high genetic differentiation were detected (*F*_ST_ > 0.9, Figure 4). The genes located in these regions were annotated using Annovar, and 981 protein-coding genes were detected. Based on the KEGG enrichment analysis, numerous genes were enriched in metabolism pathways, such as starch and sucrose metabolism, fructose and mannose metabolism, galactose metabolism, the TCA cycle, pyruvate metabolism, the insulin-signaling pathway, glycolysis/gluconeogenesis and the AMPK-signaling pathway. In addition, some genes were involved in the cell cycle, the regulation of the actin cytoskeleton, the p53-signaling pathway and apoptosis (Figure 5).

## 3. Discussion

The giant grouper is the largest coral-reef-dwelling fish, and it has been widely used for crossbreeding as the male parent. The Hulong hybrid grouper is one of the most successful hybrids, bred from the brown-marbled grouper and giant grouper. It shows the advantageous traits of both parents, and it greatly promotes the development of the grouper industry. Here, a high-quality chromosome-level genome of the giant grouper was assembled, and the genomic differences between the parents of the Hulong hybrid grouper were analyzed.

### 3.1. Genome Assembly and Evolution Analyses

The chromosomal genome of the giant grouper was constructed using Illumina and PacBio sequencing and Hi-C technology. The assembled genome is 1.06 Gb in length, which is slightly smaller than Wang’s result (1.128 Gb) [4] and Zhou’s result (1.08 Gb) [13]. Compared to the other two published genomes, the new genome showed a higher quality [4,13]. For example, the contig length N50 of the new genome (24.33 Mb) is far higher than those of the other two genomes (119.9 kb and 1469.4 kb, respectively); the contig number of the new genome (170) is far less than those of the other two genomes (27,630 and 3207, respectively); the scaffold number of the new genome (69) is also far less than those of the other two published genomes (4207 and 3187, respectively); and the chromosomal anchor of the new genome is better than those of the other two published genomes. A high mapping ratio (98.21%) of Illumina clean reads against the assembled genome was observed, 438 (95.63%) CEGs and 188 (75.81%) highly conserved CEGs were present in the assembled genome, 4463 (97.36%) complete BUSCOs were identified and 4357 (95.05%) of them were single-copy BUSCOs. These results indicate the high quality of the genome. Recently, the brown-marbled grouper genome was published, which provided essential resources for the analysis of the genetic differences between the parents of the Hulong hybrid grouper.

### 3.2. Analysis of Differences in the Genomic Structure between the Brown-Marbled Grouper and Giant Grouper

Through interspecific collinearity analysis, a significant genome synteny between the brown-marbled grouper and giant grouper was observed, while the divergence time was relatively late [11], and lots of SVs between the brown-marbled grouper and giant grouper genomes were detected. Variations were mainly distributed in introns and intergenic regions, but numerous repeat mutations were located in exons. Recent reports have shown that repeats are closely related to gene expression. For example, thousands of repeats showing regulatory effects for protein-coding genes were identified and demonstrated in *Caenorhabditis elegans* [15]. A repeat mutation located in the cold-induced autoinflammatory syndrome 1 (CIAS1) gene was associated with hypertension [16]. From our results, the protein-coding genes involved in repeat mutations were found to be involved in multiple metabolic pathways, including lipid metabolism (glycerophospholipid metabolism, fatty acid metabolism and biosynthesis), amino acid metabolism (histidine, glutamate, alanine, aspartate and glutamate metabolism), glycometabolism (insulin secretion, fructose and mannose metabolism, N-glycan metabolism) and the TCA cycle. These pathways are crucial to cellular and organismal homeostasis and play important roles in energy utilization and storage and signal transduction. Fatty acids, amino acids and carbohydrates are necessary energy substances for animals, and differences in the metabolisms between the two species may result in different levels of efficiency in the assimilation of energy. The tricarboxylic acid (TCA) cycle is a critical route for oxidative phosphorylation, which is the main source of cellular energy and participates in many metabolic pathways in organisms [17]. The mutations may influence the efficiency of ATP production. Glycerophospholipids are the most abundant phospholipids in the body, and they not only constitute biological membranes, but are also components of bile and membrane surfactants. The participation of glycerophospholipids in membrane protein recognition and signal transduction is associated with many traits and diseases, such as diabetes, obesity, neurological diseases, fatty liver and stress resistance [18]. These variations in genome sequences may give rise to differences in multiple phenotypes.

### 3.3. Analysis of Genetic Differentiation between the Brown-Marbled Grouper and Giant Grouper

Recent research has shown that the divergence time of *E. lanceolatus* and *E. fuscoguttatus* is 10.49–22.50 Myr, and their genomes possessed a significant synteny [11]. However, there was a significant differentiation between the two genomes based on SNPs: the average *F_ST_* reached 0.685. The regions with an *F*_ST_ > 0.9 were considered as demonstrating high genetic differentiation, and a total of 234 regions and 981 protein-coding genes were detected of this type. Based on the KEGG analysis, these genes were enriched in starch and sucrose metabolism, fructose and mannose metabolism, galactose metabolism, the TCA cycle, pyruvate metabolism, the insulin-signaling pathway, glycolysis/gluconeogenesis and the AMPK-signaling pathway. Numerous SNPs were located in genes related to carbohydrate metabolism. These SNPs may relate to the differences in feeding preferences and the ability to digest carbohydrates between the two grouper species under natural conditions. In a recent study, researchers explored the genetic basis of species differentiation between wolves and dogs, and they mentioned that key genes in the starch–sucrose-metabolism pathway and fat-metabolism pathway of dogs were selected to adapt to starch-based grain food in order to adapt to the farming environment of human beings in the process of evolution or domestication [19]. These genetic differences in glucose metabolism will be an important resource in future breeding works, which will be used to improve multiple production traits, such as growth, the artificial-feed-acclimation efficiency, the feed-conversion efficiency and the stocking density. Some protein-coding genes were related to the cell cycle and p53-signaling pathways, and these pathways are closely related to the regulation of the cell cycle and proliferation [20,21]. In teleost species, genes involved in pathways of the cell cycle and cell proliferation play important roles in the regulation of growth and development [22]. In rainbow trout, quantitative trait loci (QTLs) associated with bodyweight gain were detected using genome-wide association (GWA) analysis, and some genes involved in bodyweight gain were enriched in the cell cycle [23]. In broilers (*Gallus gallus*), researchers discovered that genes involved in the p53-signaling pathway are associated with the growth performance [24]. These genetic differences may be associated with the differences in body size between the brown-marbled grouper and giant grouper.

## 4. Materials and Methods

### 4.1. Sample Collection, Library Construction and Sequencing for Giant Grouper Genome Assembly

A giant grouper, *E. lanceolatus* (Figure S1), with a bodyweight of 227.0 g and a total length of 23.8 cm, was collected from Hainan Chenhai Aquatic Co., Ltd. (Hainan, China). The fish was immediately dissected after anesthesia with MS-222. White dorsal muscle tissue was sampled and immediately stored in liquid nitrogen, which was used for genomic DNA sequencing and Hi-C library construction. Moreover, ten tissues, including skin, muscle, liver, kidney, brain, intestine, fat, spleen, heart and gill tissues, were collected, immersed in RNAlater™ Stabilization Solution (Invitrogen, Carlsbad, CA, USA) and then stored at −80 °C for RNA isolation.

Total DNA was extracted from white muscle tissue with a TIANamp Marine Animals DNA Kit (Tiangen Biotech Co., Ltd., Beijing, China). The quality and quantity of the total DNA were determined via NanoDrop 2000 (Thermo Fisher Scientific Inc., Waltham, MA, USA). A paired-end sequencing library with an insert length of 350 bp was constructed using a TruSeq Nano DNA LT Library Preparation Kit (Illumina, San Diego, CA, USA). The obtained library was then sequenced using the Illumina HiSeq X Ten platform.

Genome DNA was broken into fragments using Covaris and recycled using AMpure PB beads (Pacific Biosciences, Menlo Park, CA, USA). A SMRTbell library was constructed using the SMRTbell Template Prep Kit (Pacific Biosciences, Menlo Park, CA, USA), according to the manufacturer’s instructions, and was sequenced using the PacBio Bioscience Sequel platform (Pacific Biosciences, Menlo Park, CA, USA).

Muscle samples were fixed with fresh paraformaldehyde, and then DNA–protein bonds were created. The Mbo I restriction enzyme was used to digest the DNA, and the overhanging 5′ ends of the DNA fragments were repaired with a biotinylated residue. The fragments close to each other in the nucleus during fixation were ligated, and the denatured proteins were removed. The Hi-C fragments were further sheared via sonication, and then pulled down with streptavidin beads. The library was sequenced on the Illumina HiSeq X Ten platform with PE150.

The total RNA of the 10 tissues (approximately 80 mg each) was extracted using RNAiso reagents (Takara, Dalian, China), following the manufacturer’s instructions. The quantity and quality of the RNA samples were determined using a microplate spectrophotometer (BioTek Company, Winooski, VT, USA), and electrophoresis was conducted using 1% agarose gel. The total RNA of the 10 tissues was mixed at equal amounts to generate a mixed RNA pool. RNA-seq libraries were prepared using the NEBNext UltraTM RNA Library Prep Kit (NEB, Ipswich, MA, USA), following the manufacturer’s protocols. The libraries’ qualities and quantities were measured using an Agilent 2100 Bioanalyzer (Agilent Technologies, Santa Clara, CA, USA). Finally, the libraries were sequenced using the Illumina-Hiseq 2000 platform with PE-150.

### 4.2. Genome Assembly

Raw sequencing reads were filtered using Trimmomatic version 0.38 [25] to remove low-quality data. After filtering, the clean data were used to estimate the genome size and heterozygosity using k-mer methods [26] with 21-mer.

For the PacBio-sequencing data, after removing short polymerase reads (less than 500 bp) and reads with a barcode adapter, clean data were corrected and primarily assembled using Canu version 1.8 [27]. Wtdbg was used to assemble the genome by constructing a fuzzy Bruijn graph https://github.com/ruanjue/wtdbg (accessed on 31 July 2021). Quickmerge [28] was used to merge the assemblies produced using Canu and Wtdbg, and to produce a more contiguous assembly. In simple terms, contigs from Canu were used as input queries, contigs from Wtdbg were used as references and the two types of contigs were aligned through MUMmer version 4.0 [29]. The assembled genome was polished using Pilion version 1.22 [30]. For the purposes of estimating the genome completeness, Illumina data were mapped back to the assembled genome to calculate the mapping rate using BWA v0.7.17 [31]. The genome’s integrality was verified based on the Core Eukaryotic Gene Mapping Approach (CEGMA) [32] and the Benchmarking Universal Single-Copy Ortholog (BUSCO) [33] database.

### 4.3. Pseudochromosome Construction 

The sequencing data of Hi-C were filtered to remove low-quality reads using Fastp version 0.12.6 [34]. The clean reads were aligned to the draft genome of the giant grouper using bowtie2 version 2.3.2 [35] with an end-to-end model and very sensitive parameters. The draft genome was broken into 50 Kb fragments and reassembled with the correct cluster, order and orientation of the contigs using Lachesis [36].

### 4.4. Genome Annotation

Repetitive regions of the giant grouper genome were identified via de novo and homology prediction. Transposable elements (TEs) were identified using LTR Finder version 1.05 (http://tlife.fudan.edu.cn/tlife/ltr_finder/) [37], RepeatScout v1.0.5 (http://www.repeatmasker.org) and PILER v1.0 [38]. The TEs were classified and annotated using PASTEClassifier version 1.0 using the TEdenovo pipeline [39]. Coding sequences were removed from the predicted repeat sequences through alignment to the SwissProt database using blastx with an e-value < 1 × 10^−4^, identity > 30, coverage > 30% and length > 90 bp. RepeatMasker v4.0.5 [40] was used to identify repeats based on the RepBase library version 19.06 [41] of known transposable elements (TEs).

Non-coding RNAs, including micro RNAs (miRNAs), ribosomal RNAs (rRNAs) and transfer RNAs (tRNAs), were predicted. tRNAs were predicted using tRNAscan-SE v1.3.1 [42]. microRNAs and rRNAs were predicted using Blast+ version 2.2.31 with an e-value < 1 × 10^−5^ based on the Rfam database [43]. miRNAs were predicted using Infenal version 1.1.1 [44] based on miRBase version 21 [45].

Protein-coding genes were predicted using three methods: de novo prediction, homologous-sequence prediction and RNA-seq-assisted methods. For de novo prediction, the genome without repeat regions was applied to generate gene structures using Genscan [46], Augustus version 2.4 [47], GlimmerHMM version 3.0.4 [48], GeneID version 1.4 [49] and SNAP [50]. Four fish species’ genomes and their annotation files, including tilapia (*Oreochromis niloticus*, GCF_001858045.2), zebra fish (*Danio rerio*, GCF_000002035.6), large yellow croaker (*Larimichthys crocea*, GCF_000972845.2) and Atlantic salmon (*Salmo salar*, GCF_000233375.1), were downloaded. The genome of the giant grouper was aligned to these genomes using GeMoMa [51] to obtain the exact exon, intron and splice sites [51]. RNA-seq data from ten tissues were aligned to the genome using HISAT2 version 2.0.4 [52], and assembled using Stringtie version 1.2.3 [53]. Open reading frames (ORFs) were predicted using PASA version 2.0.2 [54], TransDecoder version 2.0 https://github.com/TransDecoder/TransDecoder (accessed on 31 July 2021) and GeneMarkS-T version 5.1 [55]. The genes predicted using the three methods were merged using EVM http://evidencemodeler.sourceforge.net/ (accessed on 31 July 2021) [56].

The putative functions of predicted genes were annotated using aligned predicted genes against the NR [57], KOG [58], GO [59], KEGG [60] and TrEMBL [61] databases using Blast+ version 2.2.31.

### 4.5. Analysis of Genomic Structure between Brown-Marbled Grouper and Giant Grouper

Genomic structural variations, including deletion, insertion, tandem expansion and contraction and repeat expansion and contraction, were detected using nucmer in MUMmer 4.0.0 [62] and Assemblytics [63]. The giant grouper genome was aligned against the brown-marbled grouper genome [11], which served as a reference. The genes involved in variations were annotated using Annovar [64], with a parameter of neargene 2000.

### 4.6. Analysis of Genetic Differentiation

A total of 24 tail fins of 12 brown-marbled groupers (*E. fuscoguttatus*) and 12 giant groupers (*E. lanceolatus*) were used for genomic DNA isolation. Total DNA was extracted using a TIANamp Marine Animals DNA Kit (Tiangen Biotech Co., Ltd., Beijing, China). The quality and quantity of the total DNA were determined using a NanoDrop 2000 (Thermo Fisher Scientific Inc., Waltham, MA, USA). Paired-end sequencing libraries with an insert length of 350 bp were constructed using the Illumina Truseq DNA PCR free Library Preparation Kit (Illumina, San Diego, CA, USA). The obtained libraries were then sequenced using the Illumina HiSeq X Ten platform.

The raw data were filtered and trimmed using Trimmomatic [25]. Clean reads from each sample were aligned to the assembled genome using BWA. SNPs were called using BCFtools (version 1.10.2) [65] with mpileup, call and filter programs (https://github.com/samtools/bcftools). SNPs with a depth of <8, minor allele frequency (MAF) of <0.05, >20% missing positions, a minimum quality of <30 and a number of alleles of >2 were removed using VCFtools (version 0.1.16) [66]. The *F*_ST_ values were calculated using VCFtools with parameters including a window size of 100 Kb and an *F*_ST_ window step of 10 Kb.

## Data Availability

The raw data of the genome assembly were deposited in the Genome Sequence Archive (GSA): the project access number is PRJCA003546. The access numbers of the Illumina data for the survey, gene structure prediction and Hi-C are CRX172207, CRX166582 and CRX166581, respectively, and the access number of the PacBio data is CRX166583. The Illumina data of the resequencing of the brown-marbled grouper and giant grouper were deposited in the GSA with project access number PRJCA016709 and data access number CRA010914.

## Supplementary Materials

The supporting information can be downloaded at https://www.mdpi.com/article/10.3390/ijms241512007/s1.
