# Supplementary material for: Assembly of Genome and Resequencing Provide Insights into Genetic Differentiation between Parents of Hulong Hybrid Grouper (Epinephelus fuscoguttatus ♀ × E. lanceolatus ♂)
Source: Int J Mol Sci. 2023 Jul 26;24(15):12007. doi: 10.3390/ijms241512007 (PMC10418399; doi:10.3390/ijms241512007)
Supplement: Supplementary file 1 [file ijms-24-12007-s001.zip › ijms-2465881-supplementary.pdf]

Supplementary Materials

Assembly of Genome and Resequencing Reveals the Genetic Differences in Growth Traits between Parents of Hulong Hybrid Grouper（*Epinephelus fuscoguttatus*♀× *E. lanceolatus*♂）

Yang Yang [1,3], Leilei Zeng [1], Tong Wang [1], Lina Wu [1], Xi Wu [1], Junhong Xia [1,2] Zining Meng [1,2,*] and Xiaochun Liu [1,2,*]

1 State Key Laboratory of Biocontrol, Institute of Aquatic Economic Animals and Guangdong Provincial Key Laboratory for Aquatic Economic Animals, Life Sciences School, Sun Yat-sen University, Guangzhou 510275, People's Republic of China

2 Southern Laboratory of Ocean Science and Engineering, Zhuhai 519000, People's Republic of China

3 Key laboratory of tropical marine fish germplasm innovation and utilization, Ministry of Agriculture and Rural Affairs, Sanya 570000, People's Republic of China

*Correspondence: mengzn@mail.sysu.edu.cn (Z.M.); lsslxc@mail.sysu.edu.cn (X.L.)

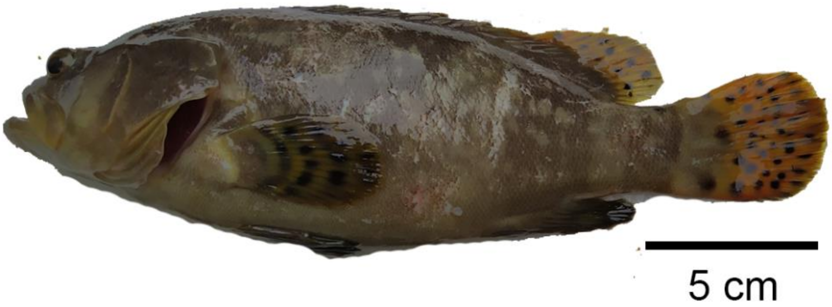

Species	Weight(g)	Total length(cm)	Body length(cm)	Body thick(cm)	Body depth(cm)
Giant grouper	227	23.8	20.6	4	7

Figure S1. Giant grouper used for genome assembly

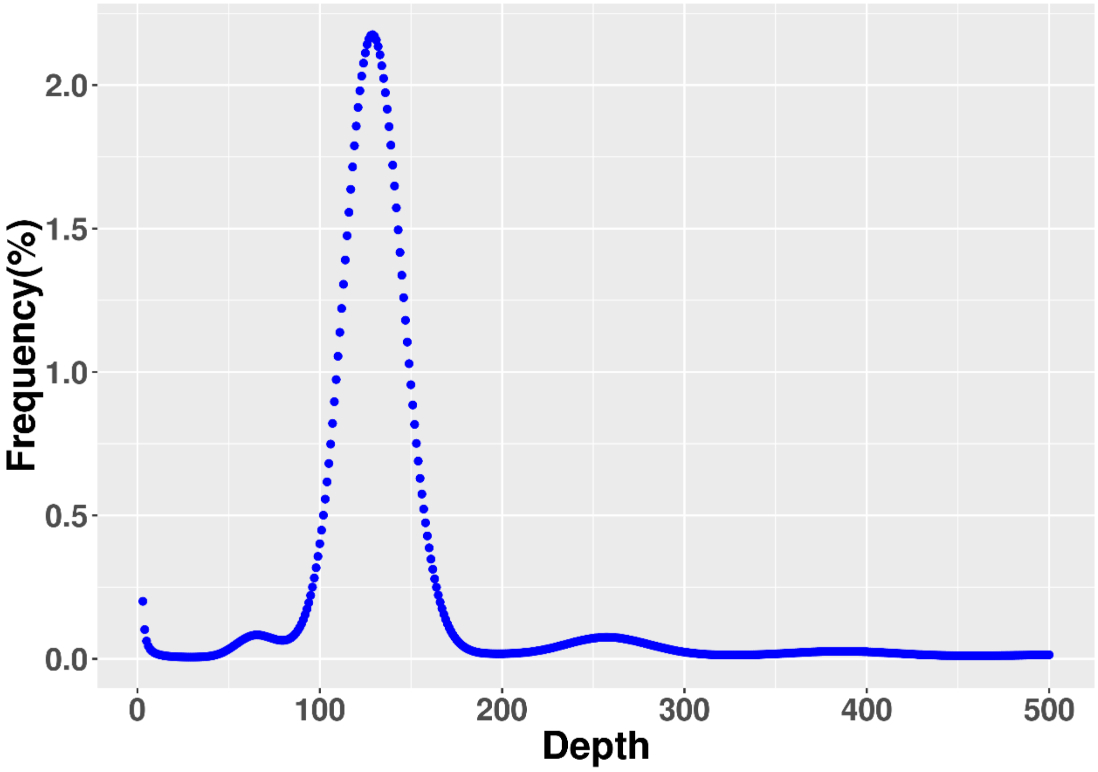

Figure S2. Distribution of K-mer of giant grouper genome

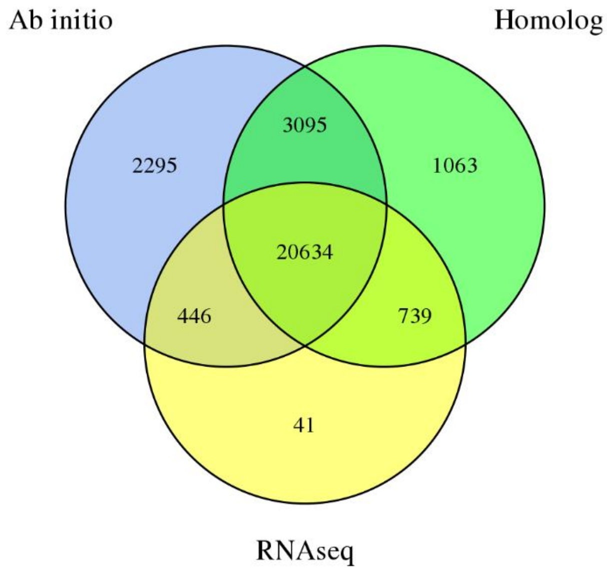

Figure S3. Prediction of gene structures in giant grouper.

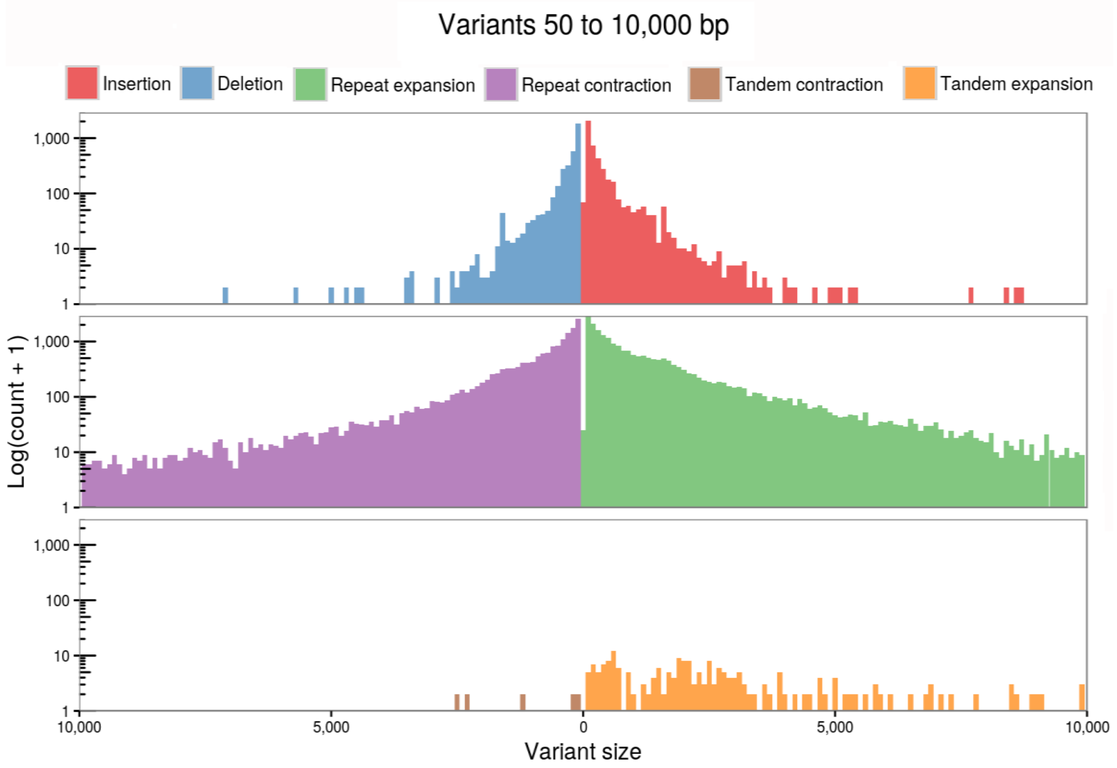

Figure S4. Variant size histogram of structural variation of genomes between brown-marbled grouper and giant grouper based on Assemblytics software. Red indicated insertion, blue indicated deletion, purple indicated repeat contraction, green indicated repeat expansion, brown indicated tandem contraction, orange indicated tandem expansion

Table S1. Statistics of sequencing data

Sequence data	Reads number	Depth	Data(Gb)	Reads length N50	Mean reads length	Max reads length	Q20(%)	Q30(%)
Illumina data	565,534,276	~156.8	169.44	150	150	150	>96.99	>92.27
Pacbio data	6,278,944	~126.3	134.42	29,899	18,676	224,633	-	-
Hi-C data	355,164,432	~98.49	106.37	150	150	150	97.18	92.45
RNA-seq	34,325,735	~9.49	10.27	150	150	150	94.26	49.73

Table S2. Mapping ratio of Illumina data in assembled genome

Data	Total_reads	Mapped_reads	Mapped (%)	Properly mapped reads	Properly mapped (%)
Illumina data	1133890136	1126054410	99.31	1110876924	98.21

Table S3. Estimation of genome integrity using CEGMA

Species	Number of 458 CEG present in assembly	Persentage of 458 CEGs present in assemblies	Number of 248 highly conserved CEGs present	Persentage of 248 highly conserved CEGs present
E. lanceolatus	438	95.63%	188	75.81%

CEG: Core Eukaryotic Genes

Table S4. Estimation of genome integrity using BUSCOs

Complete BUSCOs(C)	Complete and single-copy BUSCOs(S)	Complete and duplicated BUSCOs(D)	Fragmented BUSCOs(F)	Missing BUSCOs(M)	Total Lineage BUSCOs
4463 (97.36%)	4357 (95.05%)	106 (2.31%)	32 (0.70%)	89 (1.94%)	4584

Table S5. Chromosome statistics of giant grouper

Group	Cluster number	Cluster length	Order number	Order length
LG01	1	53717421	1	53717421
LG02	3	52981806	3	52981806
LG03	7	50638000	6	50599631
LG04	6	49470489	5	49440007
LG05	14	46272842	13	46246732
LG06	4	47475930	3	47439960
LG07	2	47770140	2	47770140
LG08	3	47285329	3	47285329
LG09	2	45121319	2	45121319
LG10	7	47926139	6	47902824
LG11	3	45206198	3	45206198
LG12	2	45448059	2	45448059
LG13	4	44756657	4	44756657
LG14	7	43992647	6	43946393
LG15	18	44813145	17	44746111
LG16	11	43436928	10	43394977
LG17	4	42591418	4	42591418
LG18	2	41752464	2	41752464
LG19	14	43502959	14	43502959
LG20	1	40025893	1	40025893
LG21	8	40162027	8	40162027
LG22	1	38313779	1	38313779
LG23	12	37424970	10	37294338
LG24	9	23505437	7	23395339
Total(Ratio %)	145(81.46)	1063591996(99.96)	133(91.72)	1063041781(99.95)

Table S6. The repeat statistics in giant grouper genome

Type			Number	Length	Rate(%)
ClassI	Total		107239	77502878	7.28
	DIRS		2137	2466326	0.23
	LARD		10791	22372850	2.1
	LINE		10707	20810924	1.96
	LTR	Copia	532	643790	0.06
		Gypsy	5608	8053920	0.76
		Unknown	3964	6875670	0.65
	PLE		60847	13850092	1.3
	SINE		1903	358941	0.03
	TRIM		9610	4125098	0.39
	Unknown		1140	161706	0.02
ClassII	Total		566676	199192232	18.72
	Crypton		13772	2567847	0.24
	Helitron		10810	2633884	0.25
	MITE		15498	4249395	0.4
	Maverick		3799	958672	0.09
	TIR		480765	185384850	17.42
	Unknown		42032	5803516	0.55
PotentialHostGene			30137	6593325	0.62
SSR			3074	938912	0.09
Unknown			293626	58470482	5.49
Total			1000752	322850618	30.34

Table S7. The prediction of gene structure of giant grouper genome

Method	Software	Species	Gene number
Ab initio	Genscan	-	43,741
	Augustus	-	42,438
	GlimmerHMM	-	105,259
	GeneID	-	42,768
	SNAP	-	63,722
Homology-based	GeMoMa	*Danio rerio*	24,290
		Salmo salar	27,558
		Oreochromis niloticus	25,968
		Larimichthys crocea	24,054
RNAseq	TransDecoder	-	51,189
	GeneMarkS-T	-	36,350
	PASA	-	26,499
Integration	EVM	-	28,313

Table S8. Predicted gene statistics of giant grouper genome

Items	Statistics
Gene number	28,313
Gene length (bp)	462,622,396
Average gene length (bp)	16,339.58
Exon length (bp)	75,031,797
Average exon length (bp)	2,650.08
Exon number	262,154
Average exon number	9.26
CDS length (bp)	46,241,736
Average CDS length (bp)	1,633.23
CDS number	255,349
Average CDS number	9.02
Intron length (bp)	387,590,599
Average intron length (bp)	13,689.49
Intron number	233,841
Average intron number	8.26

Table S9. Annotation of predicted genes of giant grouper genome

Annotation database	Annotated number	Percentage (%)
GO Annotation	13,067	46.15
KEGG Annotation	15,725	55.54
KOG Annotation	17,182	60.69
TrEMBL Annotation	26,692	94.27
NR Annotation	27,057	95.56
All Annotated	27,107	95.74

Table S10. Information of structural variation of genomes between brown-marbled grouper and giant grouper

Type	Length	Count	Total base (bp)
Insertion			
	50-500bp	3,649	607,234
	500-10,000bp	890	1,054,523
	Total	4,539	1,661,757
Deletion			
	50-500bp	3,082	504,285
	500-10,000bp	495	531,343
	Total	3,577	1,035,628
Tandem_expansion			
	50-500bp	23	6,749
	500-10,000bp	148	453,960
	Total	171	460,709
Tandem_contraction			
	50-500bp	2	212
	500-10,000bp	3	6,035
	Total	5	6,247
Repeat_expansion			
	50-500bp	8,526	1,977,461
	500-10,000bp	13,662	29,296,220
	Total	22,188	31,273,681
Repeat_contraction			
	50-500bp	7,360	1,680,172
	500-10,000bp	8,803	16,227,500
	Total	16,163	17,907,672
